# The Great Potential of DNA Methylation in Triple-Negative Breast Cancer: From Biological Basics to Clinical Application

**DOI:** 10.3390/biomedicines14010241

**Published:** 2026-01-21

**Authors:** Wanying Xie, Ying Wen, Siqi Gong, Qian Long, Qiongyan Zou

**Affiliations:** Department of General Surgery, The Second Xiangya Hospital, Central South University, Changsha 410000, China; x_iewanying@csu.edu.cn (W.X.); yingwen@csu.edu.cn (Y.W.); 258212108@csu.edu.cn (S.G.)

**Keywords:** triple-negative breast cancer, DNA methylation, diagnosis, treatment, predicting prognosis, drug resistance

## Abstract

Triple-negative breast cancer (TNBC), which is characterized by a lack of the estrogen receptor, the progesterone receptor, and HER2 expression, is the most aggressive breast cancer subtype and has a poor prognosis and high recurrence rates because of frequent chemotherapy resistance. As a crucial epigenetic regulator, DNA methylation modulates gene expression through aberrant methylation patterns, contributing to tumor progression and therapeutic resistance. Early diagnosis and treatment of TNBC are vital for its prognosis. The development of DNA methylation testing technology and the application of liquid biopsy provide technological support for early diagnosis and treatment. Additionally, preclinical and early-phase clinical studies suggest that epigenetic therapies targeting DNA methylation may hold promise for TNBC treatment, pending larger clinical trials. Furthermore, research on DNA methylation-based prognostic models enables personalized precision treatment for patients, helping to reduce unnecessary therapies and improve overall survival. The emerging role of DNA methylation patterns in predicting the therapeutic response and overcoming drug resistance is highlighted. In this narrative review, we integrate current research findings and clinical perspectives. We propose that DNA methylation presents promising research prospects for the diagnosis, treatment and prognosis prediction of TNBC. Future efforts should focus on translating methylation-driven insights into clinically actionable strategies, ultimately advancing precision oncology for this challenging disease.

## 1. Introduction

The incidence of breast cancer (BC) among women has been steadily increasing in recent years. From 2015 to 2019, there was an annual increase of approximately 0.6% to 1% in the number of BC cases. It has emerged as the most prevalent malignancy among women and ranks second as a leading cause of cancer-related death, following lung cancer [1,2]. On the basis of molecular markers, such as the estrogen receptor (ER), progesterone receptor (PR), human epidermal growth factor receptor 2 (HER2), and Ki-67, BC can be divided into three subtypes: hormone receptor-positive (HR+), HER2-positive (HER2+), and TNBC. TNBC accounts for approximately 15% to 20% of all BC cases [3,4].

TNBC is HER2-negative, with immunostaining showing less than 1% ER and PR expression. It primarily affects young premenopausal women. Because TNBC patients are the only BC subtype lacking receptor markers, endocrine therapy and targeted drug therapy are not effective. Moreover, compared with other types of BC, TNBC, as a bioaggressive tumor, has the worst prognosis, with fewer than 30% of patients achieving complete remission, and the recurrence rate and mortality are higher. Moreover, TNBC patients are more prone to developing chemotherapy resistance, which has become a major obstacle to effective treatment [5,6,7]. The diagnosis and treatment of TNBC are not yet adequate, in contrast to other BC subtypes. Thus, more effective strategies for the management of TNBC are urgently needed.

Epigenetic mechanisms influence cancer development in multiple ways and have attracted widespread research interest among researchers. Currently, the use of technologies and tools based on epigenetic mechanisms plays an important role in tumor diagnosis and treatment. As an important epigenetic mechanism, DNA methylation plays a crucial role in the diagnosis and treatment of many cancers, especially hematologic malignancies, because of its stability, universality, early stage and compatibility with liquid biopsy [8]. As with patients with other cancers, patients with TNBC also show alterations in DNA methylation. In this review, we discuss DNA methylation approaches for TNBC diagnosis, treatment and prognosis prediction and further explore its critical role in the development of drug resistance in TNBC patients.

## 2. DNA Methylation

### 2.1. DNA Methylation Is Important for Gene Expression

Gene expression is regulated by both genetic information and epigenetic mechanisms. A key characteristic of epigenetic marks is their heritability, indicating that daughter cells retain the epigenomic pattern through cell division [9]. Epigenetic changes are related to various cancers [10]. In this chapter, we focus on introducing a primary repressive epigenetic mechanism, DNA methylation. The addition of a methyl group (-CH3) to the DNA cytosine (C) base within the CpG dinucleotide to form 5-methylcytosine is a process known as DNA methylation. In the 1980s, studies revealed that DNA methylation plays important roles in gene regulation and cell differentiation. Researchers have confirmed that DNA methylation is a primary repressive epigenetic mechanism involving direct chemical modification of DNA [11,12]. As an important epigenetic mechanism, DNA methylation plays a crucial role in silencing transposable elements (TEs), regulating tissue-specific gene expression, genomic imprinting, and X chromosome inactivation in mammals [13].

### 2.2. Regulatory Programs of DNA Methylation

DNA methylation is dynamically regulated by the writers, erasers and readers of DNA methylation, which are interrelated and work together to maintain methylation homeostasis. The balance between these processes is disrupted in the context of inflammation, ageing, and cancer.

**Figure 1:**
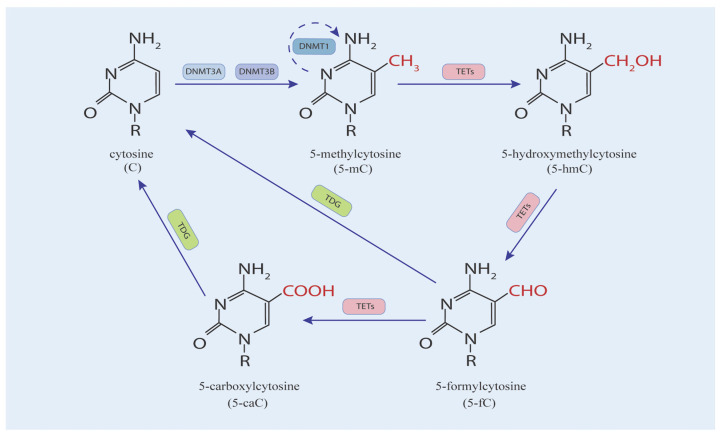
Starting from cytosine (C), DNMT3A and DNMT3B catalyse methylation to form 5-methylcytosine (5-mC). TET enzymes further modify 5-mC to 5-hydroxymethylcytosine (5-hmC), 5-formylcytosine (5-fC), and 5-carboxylcytosine (5-caC). TDG is involved in the demethylation process, converting 5-mC back to cytosine.

The addition of -CH3 during DNA methylation is dependent mainly on DNA cytosine methyltransferases (DNMTs) (Figure 1). Two families of DNMTs in this process are DNMT1 and DNMT3 proteins, which are responsible for the establishment and maintenance of DNA methylation, respectively [14,15]. DNMT3 causes de novo methylation, including that of DNMT3A and DNMT3B. DNMT1 maintains methylation following DNA replication [16].

DNA demethylation can occur in both active and passive ways. In a passive way, maintenance of DNMT1 or its associated co-factor UHRF1 weakens 5-mC by means of deactivation or nuclear exclusion over successive replication cycles. The active DNA demethylation process is regulated by ten-eleven translocation enzymes (TETs, including TET1, TET2, and TET3) [17,18]. Two sites of 5-mC, at the amino and methyl groups, can be modified chemically. TET enzymes oxidize 5-mC by adding a hydroxyl group to the methyl group, converting it to 5-hydroxymethylcytosine (5-hmC). Thus, the levels of 5-mC and 5-hmC are positively correlated [19]. 5-hmC is a recognized biomarker of malignant transformation, and reduced 5-hmC is present in a variety of cancers [20]. TETs also oxidize 5-mC to 5-carboxylcytosine (5-caC) and 5-formylcytosine (5-fC). The base excision repair (BER) protein thymine DNA glycosylase (TDG) can recognize and efficiently excise 5-caC and 5-fC oxidation products [21]. TETs and DNMTs work together to regulate DNA methylation via enzymatic activity with opposite effects. The balance between them has a crucial effect on the tumor [22].

### 2.3. The Location of DNA Methylation and CpG Islands

DNA methylation patterns are established through a tightly regulated developmental program. Following fertilization, most of the methylation from gametic DNA is removed through a process called the erasure process. The result is the formation of an epigenetic ground state [8]. Interestingly, at the time of implantation, DNA methylation usually occurs in CpG dinucleotides, except for unmethylated CpG islands. CpG islands are regions that are rich in CpG, with 60% of the protein-coding gene promoters [12]. However, notably, approximately 40% of human gene promoters do not contain CpG islands [23]. It has been demonstrated that the functions of CpG islands in human genes are not exactly the same, even within the same promoter region. Taken together, identifying the exact location of CpG islands in the clinic is important for the study of DNA methylation biomarkers [24].

## 3. The Relationship Between DNA Methylation and Disease

### 3.1. Aberrant DNA Methylation in Disease

DNMTs play crucial roles in the regulation of methylation in mammals. Abnormal changes in DNMTs are often closely associated with the occurrence and progression of tumors. DNMT1 expression is elevated in various tumor cells, including those of pancreatic cancer, gastric cancer, and lung cancer. Mutations in DNMT3A are frequently reported in hematologic malignancies and are typically associated with poor prognosis. DNMT3B, which is involved in the silencing of TSG, is overexpressed in a small subset of tumors [25]. Furthermore, studies have shown that the expression of DNMTs is upregulated in patients with TNBC, accompanied by TSG hypermethylation [26]. DNMT1 and DNMT3A overexpression is linked to shorter overall survival in patients with TNBC [27].

**Figure 2:**
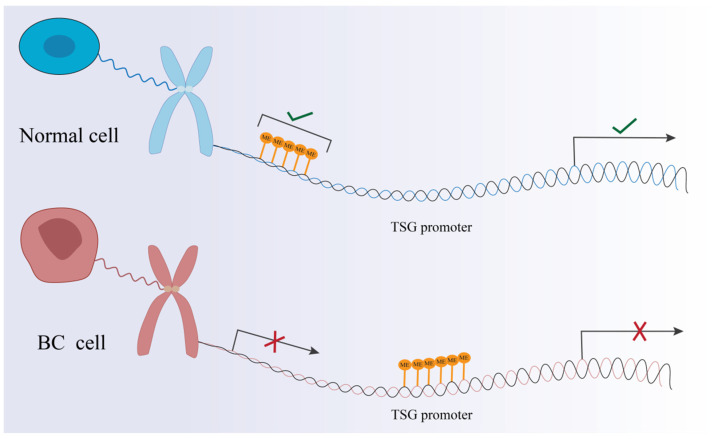
Hypermethylation of the TSG promoter region in breast cancer cells leads to incorrect transcription of genetic information on DNA.

Aberrant DNA methylation patterns are often associated with human diseases, including cancer [28,29]. Genome-wide hypomethylation occurs in tumor cells, which are distinct from normal cells. The first noticeable change in hypomethylation is the reduction in 5-mC. Furthermore, DNA hypomethylation can lead to chromosomal instability, resulting in abnormal gene expression [30]. More strikingly, hypermethylation is more frequent. Hypermethylation of the promoter region is the reason for reduced gene expression. In particular, the hypermethylation of CpG sites results in the silencing of tumor suppressor genes. The results include uncontrolled cell proliferation and oncogenesis with changes in gene regulation. Several studies raise the possibility that the combination of the proteins and the methylated groups within the chromodomain prevents the effect of activated oncogenes [31,32]. When the tumor suppressor gene (TSG) promoter region is hypermethylated, the chromatin structure changes and transforms into heterochromatin (Figure 2). Because transcription factors do not properly bind to transcription start sites and promoters, the genetic information on DNA cannot be transcribed properly. This also causes gene silencing. Hypermethylation of the TSG promoter region has been reported in numerous cancers [33].

The effect of DNA methylation is related to the location of CpG sites and disease context. The regulatory effect of DNA methylation on gene expression is reversible [34]. Given its reversibility and regulatory role, DNA methylation holds significant promise as a biomarker for the diagnosis, treatment, and prognosis prediction of various cancers.

### 3.2. Characteristic DNA Methylation Patterns in TNBC

DNA methylation patterns are primarily characterized by genome-wide hypomethylation as well as regional hypermethylation of specific genes in TNBC. The hypomethylation of oncogenes leads to their aberrant activation, which not only promotes tumor initiation and progression but also activates genes required for metastasis. By silencing growth regulatory genes, hypermethylation causes dysregulated cell proliferation [35,36]. Next, we discuss the methylation changes in TNBC.

#### 3.2.1. Hypomethylation

Jiang et al. classified TNBC into four subtypes: (1) luminal androgen receptor (LAR), (2) immunomodulatory, (3) basal-like immune-suppressed, and (4) mesenchymal-like [37]. Studies have shown that mesenchymal-like subtype tumors also exhibit global DNA hypomethylation [38]. As shown in Table 1, global DNA hypomethylation is significantly associated with TNBC.

Mendaza et al. [39] used a methylation array to compare the methylation levels in TNBC and nonneoplastic breast tissue samples (Table 1). They reported 27 and 16 probes, which recognized 17 and 10 hypermethylated and hypomethylated genes, respectively. By comparing the methylation patterns of TNBC and other subtypes of BC, the authors reported that a disintegrin and metalloprotease 12 (ADAM12) was specifically hypomethylated in TNBC. ADAM12 is a cell surface metalloproteinase and is upregulated in TNBC. It can induce the transformation of epithelial cells into mesenchymal cells and promote the invasion and migration of tumor cells [40,41]. This is not seen in other types of BC, and the authors suggested that the upregulated expression of ADAM12 in TNBC is partly caused by DNA hypomethylation. When ADAM12 was silenced in tumor cells, TNBC cell migration and proliferation were reduced, and cell sensitivity to doxorubicin was increased [39].

**Table 1:** Gene-specific hypomethylation in triple-negative breast cancer.

Author (Year)	Epigenetic Alterations	Gene	Samples	Methods	Potential Clinical Utility	Reference
Saioa Mendaza et al. (2020)	hypomethylation	ADAM12	Blood, tumor tissue	Bisulfite sequencing	Prognostic biomarker, therapeutic target	[39]
Guangcun Cheng et al. (2016)	hypomethylation	TIMP-1	Blood	Bisulfite sequencing	Prognostic biomarker, therapeutic target	[42]
Chunxiao Liu et al. (2021)	hypomethylation	LINC00511	tumor tissue	NGS	Prognostic biomarker	[43]
Saioa Mendaza et al. (2021)	hypomethylation	FLJ43663, PBX Homeobox 1 (PBX1), and RAS P21 protein activator 3 (RASA3)	Blood, tumor tissue	Bisulfite sequencing	diagnostic biomarker	[44]
Chen Chen (2021)	hypomethylation	CT83	Blood, tumor tissue	--	therapeutic target	[45]
Mehdi Manoochehri et al. (2023)	hypomethylation	LINC10606 and TBCD/ZNF750	Blood	ddPCR	diagnostic biomarker	[46]

Metallopeptidase inhibitor 1 (TIMP-1) regulates cell proliferation and survival, as well as tumor invasion and migration. Studies have shown that TNBC patients exhibit hypomethylation of the TIMP-1 promoter, resulting in a significant increase in TIMP-1 levels. An increase in the TIMP-1 level is related to poor prognosis in TNBC patients. Therefore, it has the potential to be a biomarker and therapeutic target for the poor prognosis of TNBC patients [42]. DNA hypomethylation leads to high expression of LINC00511, which is associated with invasion, metastasis, and poor prognosis in BC patients. These findings are supported by experimental evidence from Liu et al. [43].

In addition to the well-characterized alterations in ADAM12, TIMP-1, and LINC00511, researchers have also conducted novel explorations. Additionally, Mendaza et al. [44] measured the methylation levels of tissue samples from 51 TNBC patients and 16 nontumor patients by pyrosequencing. They demonstrated that the hypomethylation of FLJ43663, PBX homeobox 1 (PBX1), and RAS P21 protein activator 3 (RASA3) genes remains largely unchanged at any time in TNBC. The authors believe that these three genes can be used in the diagnosis of TNBC, especially in the early stage. One study revealed that hypomethylation of the CT83 gene caused its abnormal activation and overexpression in TNBC patients. CT83 may be a potential therapeutic target for TNBC patients [45]. LINC10606 and the TBCD/ZNF750 gene regions are hypomethylated in TNBC patients [46].

#### 3.2.2. Hypermethylation

The O6-methylguanine-DNA-methyltransferase gene (MGMT) encodes related proteins that clear mutagenic and cytotoxic adducts through DNA repair (Table 2) [47]. Al-Moghrabi et al. [48] examined MGMT methylation levels in 1534 women in Saudi Arabia and reported that MGMT promoter methylation was detected in 11% of patients with TNBC. In conclusion, the authors suggested that the use of MGMT as a biomarker may contribute to the early diagnosis of young patients with TNBC.

**Table 2:** Gene-specific hypermethylation in triple-negative breast cancer.

Author (Year)	Epigenetic Alterations	Gene	Samples	Methods	Potential Clinical Utility	Reference
Nisreen Al-Moghrabi et al. (2024)	hypermethylation	MGMT	blood	MS-PCR	diagnostic biomarker	[48]
Karolina Prajzendanc et al. (2020)	hypermethylation	BRCA1	blood	MeDIP-seq	Prognostic biomarker, diagnostic biomarker	[49]
Fei Lin et al. (2023)	hypermethylation	TERT	tumor tissue	Bisulfite sequencing	prognostic biomarker	[50]
Xiaoyu Zhang et al. (2020)	hypermethylation	ABCC9, NKAPL	tumor tissue	NGS	prognostic biomarker, diagnostic biomarker	[51]
Mehdi Manoochehri et al. (2023)	hypermethylation	CDKL2, SPAG6	blood	ddPCR	prognostic biomarker, diagnostic biomarker	[46]
Mehdi Manoochehri et al. (2020)	hypermethylation	LINC00299	blood	ddPCR	diagnostic biomarker	[52]
Brandon Griess et al. (2020)	hypermethylation	SOD3	tumor tissue	Bisulfite sequencing	therapeutic target	[53]

Breast cancer susceptibility gene 1 (BRCA1), located on chromosome [17], is a typical TSG and performs the vital functions of cell cycle control, DNA repair, and homologous recombination. Several studies have shown that BRCA1 promoter hypermethylation inactivates BRCA1 gene transcription and expression, leading to the loss of the BRCA1 protein [54,55]. However, an analysis of the BRCA1 methylation status of 942 BC patients by Prajzendanc et al. [49] revealed that BRCA1 promoter hypermethylation in peripheral blood cells was an important risk factor for TNBC. To validate their findings, they included a control group of 500 participants. In addition, the risk of TNBC in the BRCA1 promoter hypermethylation group was five times greater than that in the control group. Furthermore, Khan et al. [56] corroborated these results by performing immunohistochemical analysis of BRCA1 in 81 invasive sporadic breast carcinoma (SBC) mastectomy specimens. They reported BRCA1 promoter methylation in 68% of TNBC patients, reinforcing the association between BRCA1 hypermethylation and TNBC.

Telomerase reverse transcriptase (TERT) is involved in the encoding of telomerase. TERT promoters are hypermethylated in TNBC. The TERT promoter contains four CpG sites, and hypermethylation of three of them is positively correlated with TERT expression [50,57].

The hypermethylation alterations of MGMT, BRCA1, and TERT in TNBC have been extensively studied, and research into novel methylation changes continues to advance. Zhang et al. [51] analyzed The Cancer Genome Atlas (TCGA) database in two ways: differential expression and methylation analysis. Abnormal hypermethylation of ABCC9 and NF-κB-activating protein-like (NKAPL) genes was detected in TNBC. Multivariate Cox analysis revealed that Cox models based on the ABCC9 and NKAPL genes could be used to predict the prognosis of TNBC. Furthermore, various studies have shown that ABCC9 and NKAPL have the potential to be diagnostic and prognostic markers in different cancers [58,59]. In addition, high levels of methylation of the CDKL2, SPAG6, and LINC00299 genes have been detected in the blood samples of TNBC patients [46,52]. Griess et al. demonstrated that elevated levels of DNA methylation in the SOD3 promoter (−108 and −19 from the TSS) downregulated its expression in BC, especially in luminal B subtypes. In this research, the −78 CpG site was proven to be the most important methylation site [53].

The aberrant DNA methylation patterns observed in specific genes are not isolated events but converge to drive the defining molecular and phenotypic hallmarks of TNBC. These epigenetic alterations functionally map onto core biological processes, including DNA repair deficiency, immune evasion, epithelial-to-mesenchymal transition (EMT), stemness, and metabolic reprogramming, which collectively underpin the aggressiveness and therapy resistance of this subtype. DNA repair defects and genomic instability are driven primarily by promoter hypermethylation and silencing of key genes such as BRCA1 and MGMT. Enhanced invasion, metastasis, and EMT are promoted by hypomethylation and overexpression of genes including ADAM12, TIMP-1, and LINC00511. Stemness and cellular plasticity are fostered by methylation-mediated silencing of differentiation-related genes. Furthermore, these changes modulate the immune microenvironment, as suggested by the hypomethylation-driven overexpression of ADAM12, the hypermethylation-mediated silencing of the antioxidant gene SOD3, and the altered methylation of immune-related pathway genes such as NKAPL. Together, they do not occur in isolation but are functionally interwoven to establish and maintain the aggressive features of the TNBC.

## 4. Diagnosis of TNBC on the Basis of DNA Methylation

The diagnosis of TNBC is complicated in that early TNBC manifests in a latent state, without clinical presentation [60]. Early diagnosis and treatment of TNBC are vital for its prognosis. If TNBC is diagnosed early, the prognosis significantly improves, with the 5-year overall survival rate reaching 91% [61]. Therefore, early detection plays a pivotal role in the treatment and prognosis of TNBC patients. Ultrasound and mammography are currently used to diagnose early invasive BC, and tissue biopsy is performed when necessary [62]. In addition to traditional pathological diagnosis, multigene assays (MGAs), which are based on gene expression profiling and gene mutations, are being improved considerably. However, these methods do not respond well to the early diagnosis of TNBC. During the progression of TNBC, cells undergo epigenetic changes, such as DNA methylation and histone modification. The results show that DNA methylation changes occur in the early stages of TNBC; hence, detecting methylation biomarkers is being investigated as a potential method for the early detection of TNBC.

Currently, many scientists are exploring the use of these changes as noninvasive and reliable tools for early TNBC detection. In this chapter, we discuss the newest and most comprehensive study on the early diagnosis of TNBC based on DNA methylation detection [63].

### 4.1. DNA Methylation Detection Techniques

There are three main ways to analyze DNA methylation: bisulfite conversion, enzymatic digestion and affinity enrichment-based techniques [64].

Many DNA methylation analysis techniques are based on bisulfite conversion. Bisulfite conversion is the conversion of cytosine to uracil through a series of reactions and the amplification of uracil to thymine by PCR, which may lead to DNA damage. The 5-mC produced after DNA methylation is still cytosine after PCR amplification [65,66]. In addition, bisulfite-based methods include methylation-specific PCR (MS-PCR), combined bisulfite restriction analysis (COBRA), methylation-specific high-resolution melting (MS-HRM), bisulfite sequencing and reduced representation bisulfite sequencing. Direct Sanger sequencing, bisulfite-cloning sequencing and pyrosequencing are types of bisulfite sequencing. Pyrosequencing is among the important techniques for detecting DNA methylation patterns [67].

Since the restriction enzymes MspI and HpaII can specifically recognize the CCGG sequence, the degree of methylation of the CCGG sequence was determined by restriction pattern analysis by gel electrophoresis after the use of MspI and HpaII [68]. The advantages of this approach are its simplicity and speed. However, this method cannot be used to detect methylation levels at individual sites [69]. One of these methods, methylation-sensitive restriction enzyme PCR (MSRE-PCR), targets hypermethylated regions in tumor tissues and hypomethylated regions in normal tissues. MSRE-PCR is considered an effective method for detecting tumors [70]. Methylation-sensitive multiplex ligation-dependent probe amplification (MS-MLPA) is also based on restriction enzyme technology [71].

Methylated DNA immunoprecipitation followed by sequencing (MeDIP-seq) is an affinity enrichment-based assay for DNA methylation analysis. This method offers high genome-wide coverage, particularly in regions with high CpG density. Among current techniques, MeDIP-seq evaluates the largest proportion of the genome, making it highly suitable for global methylation profiling [72].

In recent years, next-generation sequencing (NGS) has developed rapidly, and bisulfite sequencing technology has evolved from detecting target regions to whole-genome sequencing. Whole-genome bisulfite sequencing (WGBS) requires library preparation and conversion of unmethylated cytosine to uracil on the basis of bisulfite conversion. Second-generation sequencing platforms such as Illumina or Infinium HumanMethylation450K were used for sequencing, and the data were processed using bioinformatics methods [73,74]. WGBS can detect the methylation characteristics of the whole genome. WGBS is the gold standard for DNA methylation analysis, but it is expensive. In contrast, the reduced representation of bisulfite sequencing (RRBS) target genome range is reduced, the read depth is increased, and the cost is lower [75].

Droplet digital PCR (ddPCR) is a single-molecule analysis technique that wraps nucleic acids (DNA/RNA) into discrete microdroplets and Poisson. After PCR amplification, the nucleic acid content was quantitatively analyzed according to the proportion of nonfluorescent partitions. It has the advantages of high sensitivity, high accuracy and repeatability. This detection technique plays an important role in liquid biopsies [76,77].

However, each of these current methylation detection methods involves inherent trade-offs among sensitivity, specificity, and cost. In current practice, targeted approaches such as ddPCR, MS-PCR, pyrosequencing, and MS-HRM are the most clinically feasible. These methods are cost-effective, reproducible, and well-suited for validating specific methylation biomarkers (e.g., in BRCA1, MGMT promoters) in both tissue and liquid biopsy samples, supporting their integration into routine diagnostic and prognostic workflows.

In contrast, genome-wide profiling technologies remain primarily in the research domain. WGBS, RRBS, MeDIP-seq, and high-density methylation microarrays provide comprehensive methylation maps but are limited by high costs, computational demands, and the need for extensive validation. Similarly, multi-gene signatures and integrated omics models, while promising for precision oncology, are not yet standardized for clinical use. This distinction between clinically adopted targeted assays and exploratory genome-wide platforms is crucial for evaluating the immediate applicability of methylation-based diagnostics.

Therefore, the further exploration and development of DNA methylation technology is crucial for its clinical application in the diagnosis, prognosis prediction, and personalized treatment of TNBC.

### 4.2. DNA Methylation Based on Liquid Biopsy in TNBC

**Figure 3:**
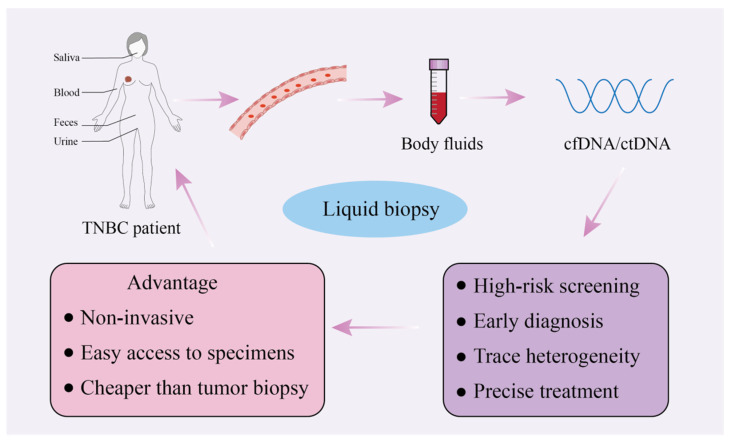
Body fluids such as saliva, blood, faeces, and urine are collected and analyzed to extract cfDNA/ctDNA. The advantages of a liquid biopsy include being noninvasive, providing easy access to specimens, and being more cost effective than traditional tumor biopsies. The applications of a liquid biopsy include high-risk screening, early diagnosis, heterogeneity tracing, and enabling precise treatment strategies.

Because the amount of tumor tissue we often obtain is small, it cannot reflect the heterogeneity of the tumor. In response to this problem, an alternative to tissue samples, called liquid biopsy, has emerged in recent years [78]. A liquid biopsy is a noninvasive screening procedure for the diagnosis of BC, in which samples are taken from saliva, urine, feces and blood [79]. (Figure 3) In particular, circulating cell-free DNA (cfDNA) and circulating tumor DNA (ctDNA) can be detected in peripheral blood samples. cfDNA is released into the bloodstream through apoptosis, necrosis, or active secretion and carries genome-wide DNA information. ctDNA is a type of cfDNA and is a small component of it [80]. As degraded DNA fragments, cfDNA from blood is highly valuable for the early diagnosis of cancer. Notably, DNA methylation patterns in cfDNA are stable and appear early in tumorigenesis, making them ideal diagnostic biomarkers [81]. As a noninvasive biopsy method, a liquid biopsy is easy to achieve and results in less trauma. In addition, detecting cancer cells in the circulating blood by specific markers can not only confirm the presence or absence of cancer but also identify specific cancer types. A liquid biopsy can detect not only the heterogeneity of tumors but also a wide range of tumors [82,83,84].

Lennon et al. [85] conducted a study called Detecting Cancer Earlier Through Elective Mutation-based Blood Collection and Testing (DETECT-A), which included 10,006 women aged 65 to 75 years with no prior history of cancer. Blood tests were performed on all participants, and PET-CT was performed on those with abnormal results. The authors suggest that blood testing facilitates early cancer detection and that blood testing could be incorporated into routine medical care as a safe and feasible test. Moreover, several studies have shown that DNA methylation-based liquid biopsies can be used for risk prediction, diagnosis, and classification of BC patients [86,87]. Liu et al. [88] conducted WGBS on 20 tumor samples and calculated the malignant cfDNA ratio of hypomethylated differentially methylated regions (hypo-DMRs). Subsequently, a model of cfMeth scores and diagnostic imaging was established. In this study, the authors suggest that a liquid biopsy combined with traditional imaging can considerably improve the accuracy of early BC diagnosis while largely avoiding needle biopsies in BI-RADS type 4 patients.

As previously mentioned, liquid biopsy technology has matured, and as a noninvasive detection method, it avoids the need for tissue biopsies in patients. By detecting methylation changes in cfDNA present in bodily fluids, particularly blood, early diagnosis and high-risk precision screening for TNBC become possible. This further underscores the tremendous potential of targeting DNA methylation for the diagnosis and treatment of TNBC patients.

### 4.3. Challenges in the Clinical Application of DNA Methylation

DNA Methylation Detection Techniques hold significant potential for application in TNBC. However, several critical issues must be addressed before these assays can be successfully integrated into routine clinical practice. First, the current landscape features a wide variety of detection techniques, and the lack of a standardized, clinically validated methodology affects the reproducibility and comparability of results. Second, the high cost of comprehensive methylation profiling, such as whole-genome sequencing, limits its accessibility in standard healthcare settings [89,90]. Furthermore, the interpretability of complex methylation signatures poses challenges for clinical decision-making. Another major challenge lies in the regulatory requirements. Regulatory agencies, including the U.S. FDA and the European EMA, mandate rigorous test validation. Such validation, which encompasses analytical validity, clinical validity, and clinical utility, is a prerequisite for approval in routine clinical use [91].Therefore, addressing these challenges is crucial for realizing the full clinical potential of DNA methylation analysis.

### 4.4. Diagnostic Biomarkers of TNBC

The rapid development of DNA methylation detection technologies has provided strong support for understanding the epigenetic mechanisms of TNBC. In the previous sections, we discussed the methylation changes identified in TNBC, with some methylation changes recognized as having the potential to serve as diagnostic biomarkers for TNBC. The sample can be not only breast tissue but also biological fluids. However, the use of the liquid biopsy has expanded our sample types beyond just breast tissue.

Studies have shown that changes in these biomarkers can also be detected not only in breast tissue but also in other bodily fluids. For example, Manoochehri et al.’s [46] study included tissue and blood samples from TNBC patients and normal subjects. Genome-wide methylation analysis of tissue samples revealed differential methylation regions (DMRs) between TNBC and normal breast tissue. After the optimal DMRs were selected, the ddPCR technique was used for evaluation of blood samples. The results revealed that methylation of the SPAG6, LINC10606, and TBCD/ZNF750 genes significantly differed between TNBC patients and the control group. Manoochehri et al. [52] performed ddPCR on blood samples from 154 TNBC patients and 159 normal subjects. This study suggests that hypermethylation of the LINC00299 gene can be used as a diagnostic marker for early detection of TNBC in young women.

Research has demonstrated that BRCA1 promoter hypermethylation may serve as a novel biomarker for the diagnosis of TNBC. This change can be acquired using MeDIP-seq technology with a sample of cfDNA from BC patients [49,92]. Other biomarkers for TNBC based on methylation changes include FLJ43663, PBX1, RASA3, MGMT, ABCC9, and NKAPL [44,48,51].

Research on DNA methylation biomarkers in TNBC has progressed, and many scientists have suggested that DNA methylation should be included in a BC risk prediction model. However, the sample origin, sample collection time, DNA methylation detection methods, and clinical feasibility still need further study [93]. In the future, how to accurately diagnose TNBC by DNA methylation is a problem that needs to be solved. In this regard, we agree with Kim et al. [94], who emphasized the importance of establishing a robust association between multisite DNA methylation patterns and TNBC. These sites need to be identified through many clinical and prospective studies.

## 5. The Potential of DNA Methylation in the Treatment of TNBC

It is well known that cancer patients are typically treated with chemotherapy, immunotherapy, neoadjuvant chemotherapy (NAC), and targeted therapy [95].The treatment of TNBC patients includes surgery, adjuvant chemotherapy, and radiotherapy. However, some patients were not diagnosed in time, and they did not receive operations in time. Thus, TNBC patients are usually treated with anthracycline and taxane chemotherapy, but the prognosis is poor, and the recurrence rate is high. DNMT inhibitors and PARP inhibitors have great potential in the epigenetic therapy of TNBC.

### 5.1. DNMT Inhibitor

TNBC patients have DNMT mutations that lead to abnormal methylation changes. Owing to the reversible nature of this process, drugs that target DNMT could be potential treatments in the future. DNMT inhibitors have proven useful for targeting epigenetic regulators, resulting in a reduction in DNA methylation levels. Additionally, DNMT inhibition can reverse the EMT, thus reducing the aggressiveness and metastatic potential of TNBC cells. Overall, DNMT inhibitors significantly suppress TNBC cell proliferation and represent a potential epigenetic therapeutic approach [96,97].

On the basis of these alterations, many DNMT inhibitors, such as decitabine (5-aza-2′-deoxycytidine) and azacytidine (5-azacytidine), have been developed. Decitabine is authorized by the FDA for the treatment of hematologic malignancies. However, studies indicate that the effectiveness of decitabine in hematologic malignancies often depends on high doses, which can be toxic to solid tumors. Studies have shown that the use of decitabine and azacytidine can significantly inhibit the proliferation of TNBC cells, but clinical progress remains limited [98,99,100].

Studies have shown that curcumin, as a natural bioactive compound, can treat BC by targeting DNMTs [101]. Many researchers have treated BC cells with curcumin and reported that the protein and mRNA levels of DNMT1 were significantly decreased [102,103]. Saghatelyan et al. formulated an innovative curcumin formulation, CUC-01, for intravenous administration. The formulation was prepared by dissolving curcumin in a solution of citric acid, ethanol, and the high-purity non-ionic solubilizer Kolliphor^®^ ELP (polyoxyl castor oil), thereby bypassing the limitation of poor oral bioavailability. A dose of 300 mg was infused weekly. In their clinical trial involving patients with advanced breast cancer, the combination of weekly paclitaxel (80 mg/m^2^) and this intravenous curcumin over 12 weeks yielded a significantly superior objective response rate versus paclitaxel with placebo. The combination also exhibited a favorable safety profile without introducing major new safety concerns [104].

Eribulin, a synthetic analogue of halichondrin B, is a novel microtubule inhibitor used in chemotherapy. Research indicates that eribulin may broadly influence DNA methylation in breast cancer cells, potentially through upregulation of DNMT3A/B and downregulation of DNMT1. In a study by Huang et al., eribulin-based regimens demonstrated a promising response rate and showed substantial improvement in progression-free survival (PFS) and overall survival (OS) compared to other chemotherapy options, such as platinum-based agents and nab-paclitaxel, in patients with advanced TNBC. However, these clinical benefits cannot yet be directly attributed to epigenetic modulation, and the mechanistic link remains speculative [105,106,107].

Jan et al. [108] reported that ZINC167686681, a novel DNMT inhibitor, has excellent pharmacokinetic characteristics. However, the development of ZINC167686681 is immature, and further research is needed. Flavonoids, which are widely distributed in plants, possess cytotoxic effects and have been shown to inhibit cell proliferation, induce apoptosis, and consequently reduce the risk of breast cancer. This makes them very important in the research of anticancer drugs [109]. In a study by Liang et al. [110], liquiritigenin, a natural flavonoid, controlled the aggressiveness of TNBC by decreasing DNMT activity. However, both of these novel inhibitors have been confirmed to have anticancer effects only at the BC cell level, and further preclinical experiments are needed to validate the efficacy and safety of these drugs.

DNMT inhibitors can have significant cytotoxic effects on TNBC cells. However, DNMT inhibitors are effective only in patients with DNMT1 expression [111,112].

Significant side effects accompany their tumor-suppressing effects, leading to the prohibition of DNMT inhibitors in clinical trials for solid tumors. Consequently, increasing attention has shifted towards combination therapies, such as combining DNMT inhibitors with chemotherapy, ERRα or immunotherapy [113]. In future research, addressing the side effects and resistance associated with DNMT inhibitors will remain a critical challenge.

### 5.2. Epigenetic Drugs Combined with Immunotherapy for the Treatment of TNBC

Immunotherapy, a treatment approach that stimulates the human immune system to kill tumor cells [114]. The introduction of immune checkpoint inhibitors (ICIs) targeting PD-1 and PD-L1 has shown promise in a variety of cancers. Research has shown that the immune system plays a pivotal role in the development and progression of BC. Immunotherapy, particularly immune checkpoint blockade, offers hope for BC patients, sometimes providing OS benefits. However, the immune microenvironment in TNBC often limits the efficacy of immunotherapy [115,116,117]. Epigenetic mechanisms play critical roles in the activation, differentiation, and effector functions of immune cells. Through transcriptional and metabolic reprogramming of local immune cell populations, epigenetics influences the tumor microenvironment, including immune cell composition, cytokine signaling, and immune checkpoint expression, ultimately leading to immune evasion. Epigenetic modifications, including DNA methylation, promote the expression of immunosuppressive factors by silencing genes involved in antigen presentation and immune activation [118,119]. By targeting these epigenetic mechanisms, preclinical and early clinical data suggest that combining epigenetic drugs with immunotherapy may offer a synergistic approach for treating TNBC, warranting further investigation. This combined treatment regimen can simultaneously affect tumor cells and the TME to overcome immune escape or resistance to immunotherapy. Early evidence suggests that checkpoint inhibitors combined with DNMTi are effective across various cancers, including Hodgkin’s lymphoma, BC, mesothelioma, lung cancer, and melanoma [120,121].

### 5.3. PARP Inhibitors

BRCA1 promoter hypermethylation is common in BC, especially in TNBC. Many studies suggest that BRCA1-deficient patients are sensitive to poly (ADP-ribose) polymerase (PARP) inhibitors and cisplatin therapy. PARP inhibitors target BRCA1 by exploiting genomic instability and deficiencies in DNA repair pathways, thus exerting their cytotoxic effects on tumor cells [122]. Four PARP inhibitors, namely, niraparib, rucaparib, olaparib, and talazoparib, have been approved for the treatment of BRCA-mutated BC [123]. The recent results indicated that olaparib significantly improved the survival rate and reduced the recurrence rate [124]. Muvarak et al. [125] studied the effects of combining PARP inhibitors with low-dose DNMT inhibitors in cancer treatment. The results indicated that the combination of these two inhibitors at very low doses could enhance clinical efficacy in AML patients. The role of this combination in the treatment of BC patients remains to be further explored.

### 5.4. Antibody–Drug Conjugates

Antibody-drug conjugates (ADCs) combine the potent cytotoxicity of chemotherapy with the antigen-specific targeting capability of antibodies into a single molecule [126]. Sacituzumab govitecan (SG) consists of a humanized monoclonal antibody that targets trophoblast cell surface antigen 2 (TROP2) and is linked to the DNA topoisomerase I inhibitor SN-38. Its pH-sensitive cleavable linker breaks within tumor cells, releasing the cytotoxic payload SN-38 to kill the tumor cells. TROP2 is a transmembrane glycoprotein that is highly expressed in several solid tumor types, including TNBC [127]. SG has demonstrated efficacy against TNBC in preclinical studies [128,129]. Studies have shown that decitabine can reduce the expression of DNA methyltransferase and the methylation of the TROP2 promoter, thereby significantly enhancing the antitumor activity of SG [130]. Furthermore, decitabine has also demonstrated synergistic effects with ADC drugs in multiple preclinical models of melanoma [131].

## 6. Predicting the Prognosis of TNBC Based on DNA Methylation

TNBC patients are heterogeneous, with significant differences in both response to NAC and survival among patients with metastasis. Therefore, TNBC patients cannot use a single treatment approach, and we can provide precise and individualized management for patients through prognostic prediction [132]. Moreover, emphasizing the prognosis prediction of TNBC patients helps reduce unnecessary treatments and improve their OS.

### 6.1. Biomarkers for Predicting Prognosis

Several studies have shown that aberrant methylation changes in BC patients can be used to predict treatment effects and survival outcomes. Researchers have conducted methylation detection on prognostic biomarkers to determine a patient’s response to drugs and guide the treatment of TNBC patients. The development of valuable prognostic biomarkers is highly important for determining the treatment, prognosis, and survival rate of patients [133].

DNA methylation-based biomarkers can be used to predict the overall prognosis of TNBC. For instance, Cheng et al. [42] analyzed the methylation status of TIMP-1 in the plasma of TNBC patients using bisulfate sequencing. The results revealed that TIMP-1 expression is higher in TNBC than in non-TNBC, and patients with high expression have a poorer prognosis. The authors suggest that TIMP-1 can serve as a prognostic marker for TNBC. Mendaza et al. [39] reported that ADAM12 is expressed in the plasma and paracancerous tissues of TNBC patients and that hypomethylation of ADAM12 suggests a poor prognosis.

Certain prognostic biomarkers can also be used to predict the treatment response of patients with TNBC, thus guiding physicians in providing personalized treatment while reducing unnecessary interventions and alleviating the burden on patients. Multiple studies have indicated that in patients with nonmetastatic TNBC who receive anthracycline-based adjuvant chemotherapy, outcome can be predicted by assessing the methylation status of PITX2 in tumor tissues [134]. Hypermethylation of PITX2 is related to poor outcomes in TNBC patients treated with anthracycline [135]. The methylation status of CDKL2 can also be used to predict the response to chemotherapy in TNBC patients [46]. TERT promoter hypermethylation could predict the response to immune checkpoint inhibitors and TERT inhibitors [50].

The development of prognostic biomarkers holds great significance for the treatment of TNBC patients. Moreover, with the advancement and maturity of liquid biopsy technology, dynamic and real-time monitoring of patients is becoming increasingly feasible.

### 6.2. Establishment of Prognostic Model for TNBC

Currently, researchers are continuing to study and develop predictive models for BC. These models assist doctors in predicting disease progression and treatment response in patients, thus enabling personalized precision medicine. Below, we introduce the development and clinical significance of the four predictive models (Table 3).

To establish a robust and accurate model of BC prognosis prediction based on DNA methylation, Wu et al. [136] collected BC samples from The Cancer Genome Atlas (TCGA) database and methylation information from the UCSC Cancer Browser. They subsequently identified potential methylation sites associated with OS outcomes from 21,122 sites and used 166 possible prognostic methylation sites to define distinct DNA methylation-based molecular subtypes. The model could serve a purpose in biomarker detection, diagnosis, and prognostic prediction.

Gao et al. [137] analyzed methylation data obtained from the TCGA database. They utilized the LASSO method to construct a 5-DMS signature, a prognostic model for TNBC. This model predicts both disease-free survival and OS excellently and has the potential to guide personalized treatment for TNBC patients.

Peng et al. [138] identified CpG sites that were significantly correlated with OS using univariate Cox proportional hazards regression analysis. They then employed LASSO Cox regression analysis to refine the selection, narrowing it to 15 key CpG sites and developing a risk score model. This model was rigorously validated across various datasets. Ultimately, they constructed a nomogram incorporating the CpG-based signatures along with TNM staging to predict the 1-, 3-, and 5-year OS rates for patients with TNBC.

In addition to models predicting patient OS, some models can predict patient responses to drugs. Given the significance of NAC for TNBC patients, Pineda et al. [139] developed a model that can predict the patient response to NAC. They reported that the FERD3L and TRIP10 genes were related to the prognosis of TNBC patients with NAC. They subsequently developed a two-gene prediction model based on the FERD3L and TRIP10 genes, which can accurately predict the pathological complete response (pCR) in TNBC patients.

In summary, these four models have undergone independent validation to ensure their reliability and generalizability. Their use can not only aid in the development of personalized and precise treatment plans but also help reduce the metastasis and recurrence of TNBC. By predicting patients’ responses to NAC, unnecessary treatments and related side effects can be minimized. While these models demonstrate strong predictive power, their clinical applicability depends on further validation with larger TNBC patient cohorts.

## 7. The Crucial Role of DNA Methylation in TNBC Drug Resistance

### 7.1. DNA Methylation Leads to Drug Resistance in TNBC

Chemotherapy resistance is an extremely challenging issue in the treatment of TNBC and directly affects patient prognosis and quality of life [140]. Multiple studies have shown that drug resistance in TNBC patients is caused by multiple factors and signaling pathways, including epigenetic mechanisms. Studies have shown that methylation changes at some sites are related to chemotherapy resistance [141].

When TNBC patients develop resistance to chemotherapy drugs, the methylation of CpG islands and CpG shores undergo various changes [142]. For instance, Shi et al. [143] used MeDIP-seq to detect methylation levels of MCF-7 (sensitive cells) and MCF-7/Taxol (resistant cells), and 55,076 differentially methylated genes (DMGs) were detected. The authors suggest that there are differences in DNA methylation patterns between resistant and sensitive BC cells. This occurs not only in BC but also in other cancers, such as colorectal cancer and ovarian cancer, where methylation occurs [144,145].

**Figure 4:**
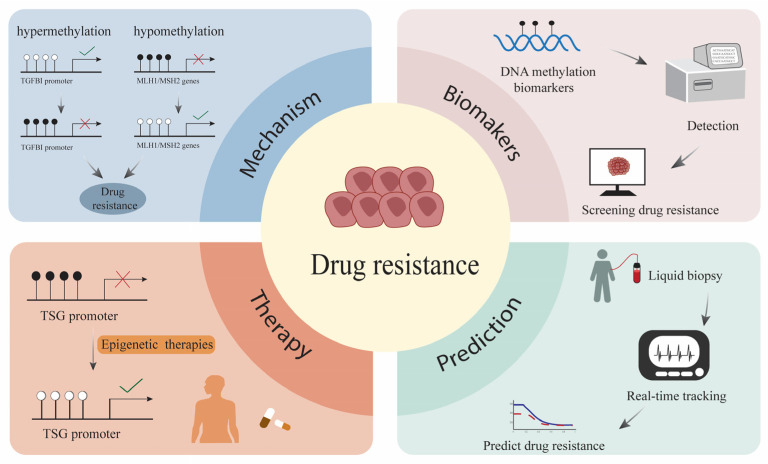
Hypermethylation of the TGFBI promoter and hypomethylation of the MLH1/MSH2 genes lead to drug resistance. DNA methylation biomarkers are used for the detection and screening of drug resistance. Epigenetic therapies target the TSG promoter to reverse drug resistance. The real-time tracking and drug resistance prediction of TNBC patients can be achieved through liquid biopsy.

Aberrant DNA methylation mediates the dysregulation of tumor suppressor genes and oncogenes, leading to drug resistance in TNBC patients [146]. For example, TGFBI is a TSG, and hypermethylation of the TGFBI promoter causes gene silencing in BC patients (Figure 4). Studies have explored the relationship between TGFBI promoter hypermethylation and drug resistance in BC patients. The results show that there is a significant correlation between them [147]. A study on 45 breast cancer samples treated with NAC and 133 untreated breast cancer samples revealed that the MLH1/MSH2 genes play a crucial role in breast cancer progression and that their hypomethylation may be associated with chemotherapy resistance [148]. The DAB2IP gene is also associated with drug resistance in TNBC patients and inhibits chemotherapy resistance [149]. The findings of these studies enhance our understanding of the mechanisms of chemotherapy resistance and provide new insights for potential detection and therapeutic targets.

### 7.2. Clinical Applications of DNA Methylation for Overcoming Chemotherapy Resistance in TNBC

Addressing chemotherapy resistance in TNBC patients is an urgent priority. In the following sections, we discuss the latest advances in DNA methylation in TNBC resistance from three perspectives: biomarkers, therapy, and prediction.

DNA methylation biomarkers are chemically stable, and current detection methods are highly advanced. Notably, changes in DNA methylation often precede the onset of chemotherapy resistance. Therefore, detecting methylation biomarkers can serve as an effective strategy for screening for drug resistance [150]. The development of methylation-based biomarkers for predicting resistance enables a more precise selection of chemotherapy drugs, allowing for personalized treatment plans tailored to individual patients [151].

One promising approach to overcoming drug resistance is targeting epigenetic regulatory factors, which play key roles in modulating DNA methylation and gene expression [152]. As described above, decitabine inhibits DNA methylation, leading to the re-expression of TSG genes. Therefore, Xiong et al. [149] proposed that targeting DNA methylation to alleviate chemotherapeutic resistance in TNBC may be a potential treatment strategy.

Research on predictive models for chemotherapy resistance in TNBC patients remains insufficiently developed [153]. Recently, dynamic monitoring of changes in ctDNA through liquid biopsies has been proposed to achieve real-time tracking of treatment efficacy and predict drug resistance in BC patients. Research on this technology can be used to guide follow-up treatment for patients and improve clinical outcomes [154].

Overall, the application of DNA methylation biomarkers for predicting resistance, epigenetic therapeutic strategies for reversing resistance, and dynamic monitoring of methylation levels for precise intervention should be extensively and thoroughly investigated. The implementation of these strategies will offer new directions for personalized treatment, enabling timely assessment of resistance risk and adjustment of treatment plans, thus improving therapeutic outcomes and patient prognosis.

## 8. Conclusions and Perspectives

As the most aggressive phenotype in BC, TNBC is characterized by poor prognosis and low survival rates, prompting a deeper exploration of this subtype. The abnormal changes in DNA methylation observed in TNBC highlight its immense potential in research on TNBC. Advances in methylation detection technology and liquid biopsy have provided strong technical support for the application of DNA methylation in TNBC patients. Epigenetic therapy presents promising research prospects for the treatment of TNBC patients. Moreover, the combination of epigenetic drugs (such as DNMTi) with immune checkpoint inhibitors holds considerable promise as a therapeutic strategy to overcome immunotherapy resistance and improve outcomes in TNBC. Predictive models incorporating DNA methylation biomarkers have the potential to optimize personalized treatment strategies, improve patient survival rates, and reduce unnecessary treatments. Chemotherapy resistance in TNBC patients is a major obstacle to treatment, and abnormal DNA methylation is among the key mechanisms underlying this resistance. Fully utilizing DNA methylation techniques will provide significant advantages in overcoming TNBC drug resistance.

However, translating these insights into routine clinical practice remains challenging due to technical variability, high costs, and the need for rigorous clinical validation. To fully realize the potential of DNA methylation in TNBC, future work should focus on several key priorities: establishing standardized and clinically validated methylation assays using uniform protocols and prospective cohorts; integrating multi-omics data to refine subtype classification and therapeutic targeting; implementing longitudinal monitoring of resistance dynamics via liquid biopsy for real-time treatment adaptation; optimizing combination epigenetic therapies, such as DNMT inhibitors with immunotherapy or chemotherapy; and developing accessible, cost-effective detection platforms to facilitate widespread adoption. By addressing these priorities, methylation-driven discoveries can be transformed into actionable clinical strategies, ultimately improving outcomes for TNBC patients.

## Figures and Tables

**Table 3:** Four prognostic models for triple-negative breast cancer.

Author (Year)	Data Sources	Methylation Sites	Functions	Ref.
Zeng-Hong Wu et al. (2021)	TCGA	166 CpG sites	OS prediction for BC	[136]
Yinqi Gao et al. (2021)	TCGA, GEO	5 DMSs	OS and disease-free survival prediction for TNBC	[137]
Yang Peng et al. (2019)	TCGA	15 CpG sites	OS prediction for TNBC	[138]
Begona Pineda et al. (2020)	TNBC samples	2 genes	Prediction of NAC response	[139]

## Data Availability

No new data were created or analyzed in this study. Data sharing is not applicable to this article.

## Abbreviations

The following abbreviations are used in this manuscript:

TNBC	Triple-negative breast cancer
BC	Breast cancer
ER	Estrogen receptor
PR	Progesterone receptor
HER2	Human epidermal growth factor receptor 2
TSG	Tumor suppressor gene
TERT	Telomerase reverse transcriptase
NGS	Next-generation sequencing
WGBS	Whole-genome bisulfite sequencing
RRBS	Reduced representation bisulfite sequencing
ddPCR	Droplet digital PCR
cfDNA	Circulating cell-free DNA
ctDNA	Circulating tumor DNA
5-mC	5-methylcytosine
5-hmC	5-hydroxymethylcytosine
5-fC	5-formylcytosine
5-caC	5-carboxylcytosine
DNMTs	DNA cytosine methyltransferases
TETs	ten-eleven translocation enzymes
BER	base excision repair
TDG	Thymine DNA Glycosylase
NAC	neoadjuvant chemotherapy
